# Development and Validation of the Parkinson’s Disease Specific Anxiety Inventory (PDSAI)

**DOI:** 10.1177/08919887251332660

**Published:** 2025-04-08

**Authors:** Nadeeka N. Dissanayaka, Dana Pourzinal, Gerard Byrne, Nancy A. Pachana, John D. O’Sullivan, Elizabeth White, Tiffany Au, Jihyun Yang, Alejandro Interian, Kailyn Rodriguez, Roseanne D. Dobkin

**Affiliations:** 1 UQ Centre for Clinical Research, 1974 The University of Queensland, Brisbane, QLD, Australia; 2 School of Psychology, 1974 The University of Queensland, Brisbane, QLD, Australia; 3 Department of Neurology, Royal Brisbane and Women’s Hospital, Brisbane, QLD, Australia; 4 Mental Health Service, Royal Brisbane and Women’s Hospital, Brisbane, QLD, Australia; 5 20061 VA New Jersey Healthcare System, Lyons, NJ, USA; 6 Department of Psychiatry, Rutgers University, Robert Wood Johnson Medical School, Piscataway, NJ, USA

**Keywords:** Parkinson’s disease, anxiety, inventory, PDSAI

## Abstract

**Background:**

Anxiety is poorly recognized and inadequately treated in persons with Parkinson’s disease (PD).

**Objective:**

The present study aimed to develop and validate a new clinical screening and research outcome measure to identify triggers and manifestations of anxiety specific to PD, the Parkinson’s disease Specific Anxiety Inventory (PDSAI).

**Method:**

Data from PDSAI derived from 172 people with PD across Australia and the United States was used to assess the reliability and validity of the inventory. Construct validity was assessed.

**Results:**

Frequency analyses revealed low rates of missing data across the 40 items. The inventory demonstrated high reliability (Cronbach’s a = 0.93, split-half = 0.68) and mid to high concurrent validity between the PDSAI and (i) Hamilton Anxiety Scale (r = 0.51), (ii) Liebowitz Social Anxiety Scale (r = 0.697) and Parkinson’s Anxiety Scale (r = 0.747).

**Conclusions:**

The PDSAI is a valid and reliable tool designed to capture PD specific triggers and manifestations of anxiety in people with PD.

## Introduction

Anxiety is a prominent non-motor symptom in Parkinson’s disease (PD) with a global average prevalence of 31%.^1^ The prevalence of anxiety in people with PD is three times higher than anxiety in older adults and presents with a diverse array of symptoms that require further characterization.^2-4^ Anxiety also detrimentally impacts activities of daily living in people with PD, even more so than motor disability.^5^ In comparison to people with PD without anxiety disorders, those with anxiety experience 50% poorer quality of life, 5 times more complications from PD treatment, and 10 times greater disability.^5^

Anxiety in PD occur with or without depression and may be chronic or episodic in presentation. In addition to well described anxiety disorders such as Panic, Generalized Anxiety, and Social Phobia, clinically significant PD anxiety may also present with unique and heterogeneous characteristics related to motor and non-motor symptoms, cognitive change, treatment complications (on-off fluctuations, dyskinesia, dose failures), and disease burden/adjustment.^4,6^ Recent review of the phenomenology of atypical anxiety disorders in PD suggested a weighted prevalence of 15% Anxiety not otherwise specified (NOS), 34% fluctuating anxiety, and 52% fear of falling.^7^ These nuances of PD anxiety, including atypical presentations, are under-diagnosed and sub-optimally treated, negatively impacting all aspects of disease management.^3,4,8^

A more nuanced understanding of PD anxiety is required to improve rates of anxiety detection and treatment initiation, as well as clinical outcomes, in people with PD. More precise measurement of anxiety will also enable clinicians to tailor the implementation of evidence-based treatments, such as Cognitive Behaviour Therapy (CBT) and Mindfulness Based Cognitive Therapy (MBCT), to meet the unique needs of each person with PD.^9,10^ As such, this study developed and validated the Parkinson’s Disease Specific Anxiety Inventory (PDSAI). This tool was designed to assist with PD-specific anxiety symptom assessment and monitoring. Unlike existing scales, the PDSAI focuses on cognitive, emotional, behavioral, and physiological triggers and manifestations of anxiety that are unique to people with PD, in an effort to facilitate screening and enable personalized care.^11,12^

## Materials and Methods

### Development of the PDSAI

A 40-item questionnaire (PDSAI) was developed based on interviews with people living with PD published in a previous study (N = 90),^2^ and clinical experience by an interdisciplinary (Neurology, Psychiatry, Geropsychology) clinician-researcher team (NND, JOS, GB, NAP) with expertise in PD anxiety, anxiety in older adults, and development of anxiety rating scales. In this way, we ensured that items selection was represented by lived experience, subject matter, and clinical experts. Based on our prior work, key concepts that emerged were as follows: (1) early detection is required for successful treatment of anxiety, (2) PD-specific triggers of anxiety need to be better understood to improve screening protocols, and (3) a more nuanced understanding of PD anxiety is required to guide treatment development, implementation, and evaluation.

**Figure 1.**
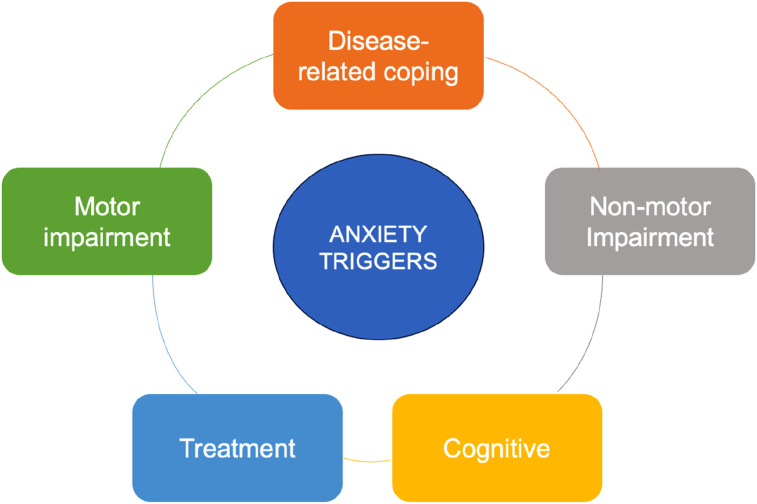
A conceptual model of triggers of Parkinson’s disease specific anxiety.

Based on expert opinion and the perspective of people with PD, a conceptual model of PD anxiety was developed to inform item content for the PDSAI and identification of triggers and manifestations of anxiety (Figure 1, Table 1). PD-specific triggers of anxiety were then identified and matched to five primary content domains related to the phenomenology of PD: (1) Disease-related coping, (2) Motor symptoms, (3) Non-motor symptoms, (4) Cognitive symptoms, and (5) Treatment (Figure 1, Table 1). Distinct manifestations of anxiety (e.g., emotional, cognitive, physiological, behavioural) associated with each domain were also considered. Forty items were ultimately derived based on this model and were reviewed by the clinician-researcher experts prior to their inclusion in the inventory. For brevity and ease of use, the questionnaire incorporated a dichotomous response set of “Yes or No,” with inquiry into experience of each item during the past week, mirroring the format of the Geriatric Anxiety Inventory (developers NP and GB).^13^ The PDSAI is available for use upon request to the corresponding author. The present manuscript is required to be cited when publishing data related to the use of the PDSAI in future.

**Table 1.** PD-specific Triggers and Manifestations of Anxiety.

Domain	Sample Triggers	Manifestations of Anxiety
Disease-related coping	PD diagnosis	Behavioral
	Insecurity about future perceived burden to others	*Poor adjustment*
	Spending time alone	*Taking extra PD medication*
	Fear of losing control	Cognitive
	Social events	*Worry*
	Travel	*Poor acceptance*
		Emotional
		*Anxiety*
		*Insecurity*
		*Fear*
Motor symptoms	Tremor, gait difficulties, postural instability, freezing of gait, fear of falling	Behavioral
		*Avoidance*
		Cognitive
		*Worry*
		Emotional
		*Anxiety*
		*Frustration*
		*Fear*
		*Embarrassment*
Non-motor symptoms	Constipation, drooling, dizziness, speech, sleep disturbance, bladder problems	Behavioral
		*Avoidance*
		Cognitive
		*Worry*
		Emotional
		*Anxiety*
		*Embarrassment*
		*Fear*
		Physiological
		*Agitation*
		*Inner unrest*
Cognitive symptoms	Memory problems, language impairment, attentional difficulties	Behavioral
		*Avoidance*
		Cognitive
		*Worry*
		Emotional
		*Frustration*
		*Embarrassment*
		*Fear*
Treatment	Deep brain stimulation and adverse effects of pharmacotherapy like on/off fluctuations, wearing off medication, impulse control disorders, and dyskinesias	Behavioral
		*Avoidance*
		Cognitive
		*Worry*
		Emotional
		*Anxiety*
		*Embarrassment*
		*Fear*

### Validation of the PDSAI

#### Participants and Setting

This international cross-sectional validation study combines data from participants (N = 172) at two sites; Brisbane, Australia (N = 107) and Lyons, New Jersey, United States (N = 65). Participants were recruited from movement disorders clinics, a US Veteran Health Administration Medical Center, and throughout the community between 2016 and 2022.

#### Inclusion and Exclusion Criteria

Participants were eligible if they had a diagnosis of idiopathic PD.^14^ Participants with other comorbid neurological disorders, dementia, bipolar and psychotic spectrum disorders, current substance abuse or dependence, and evidence of suicidal plans or intent, as well as those who were unable to complete a questionnaire by themselves or with assistance were excluded.

#### Procedure

Ethical approval was received from the University of Queensland (UQ), Royal Brisbane & Women’s Hospital and Princess Alexandra Hospital Human Research Ethics Committees (HREC) and the Veterans Health Administration (VHA) New Jersey IRB. Informed consent was collected from all participants prior to commencement of research. We used a combination of observer rated and self-report scales to validate the PDSAI.

Both sites collected demographic variables (age, sex, and disease duration) and administered the 40 item PDSAI, observer-rated Hamilton Anxiety Scale (HAM-A),^15^ observer-rated Hamilton Depression Rating Scale (HAM-D),^16^ Montreal Cognitive Assessment (MoCA),^10^ and self-report Parkinson Anxiety Scale (PAS).^17^ The PAS comprises three subscales which measure persistent anxiety (PAS-A), episodic anxiety (PAS-B), and avoidance behaviour (PAS-C).

The Mini International Neuropsychiatric Interview Plus (MINI-plus)^18^ with psychiatrist (GB) confirmation (UQ) or Structured Clinical Interview for DSM (SCID)^19^ (VHA NJ) was used to identify depressive and anxiety disorders according to the Diagnostic and Statical Manual Edition 5 criteria. The Australian site collected additional measures including the Movement Disorder Society Unified Parkinson’s Disease Rating Scale (MDS-UPDRS) to measure parkinsonism features,^20^ and the self-report Liebowitz Social Anxiety Scale (LSAS) to measure social anxiety,^21^ and Starkstein Apathy Scale (SAS) to measure apathy.^22^ The levodopa equivalent daily dose (LEDD) was derived using established methods,^23^ and all participants were assessed in the “ON” state (dopaminergic medication working optimally).

#### Statistical Analyses

Analyses were performed using SPSS v21.0 (SPSS Inc. Chicago, 2007). The threshold for missing data was set to 5%, with all measures demonstrating acceptable levels. For all analyses, ‘Not Applicable’ responses on the PDSAI were coded as ‘No’. Demographic variables were compared across sites using Kruskal-Wallis tests. Cronbach’s alpha and the split-half coefficient were calculated as measures of internal consistency and reliability, with a > 0.70 being considered acceptable.^24^ Convergent validity of the PDSAI with the HAMA, LSAS and PAS was assessed using correlation analyses. Where assumptions of normality and homogeneity of variance were met (MoCA, PAS, HAMD, SAS), Pearson’s *r* was used to assess correlations. Where skewness was greater than 1.96SD (LSAS, PAS Subscales B and C), variables were transformed using a square root transformation prior to calculating Pearson’s *r*. Where assumptions of normality and/or homogeneity of variance were violated (HAM-A), Spearman’s rho was used to assess correlation. The significance level for all analyses was set to a Bonferroni-corrected *P* < .05.

## Results

### Missing Data

The total missing data accounted for 4.4% of the dataset. However, the maximum number of missing scores for any given variable was 16%, which was above the response threshold and thus missing data analyses were performed. Little’s Missing Completely at Random (MCAR) test yielded a non-significant result, χ2 (193, 158) = 96.206, *P* > .999, indicating the data were MCAR.^25^ Due to the low quantity of missing data and its MCAR nature, cases were excluded listwise from the relevant analyses for every measure.

### Sample Characteristics

Sample (N = 172) characteristics are displayed in Table 2. The mean PDSAI score was significantly higher in people with PD with any DSM-V diagnosis of an anxiety and/or depressive disorder (N = 101; mean = 18.23; standard deviation = 8.71) compared to those without a DSM-V diagnosis of anxiety or depression (N = 46; mean = 7.33; standard deviation = 6.37).

**Table 2.** Sample Characteristics (N = 172).

Variable	Mean ± SD	Range
Age (years)	67.63 ± 8.46	41-86
Education (years)	13.12 ± 2.76	6-20
Disease duration (years)	5.31 ± 5.52	0-46
UPDRS total	37.40 ± 17.79	5-107
UPDRS-II (non-motor)	9.40 ± 6.29	1-34
UPDRS-III (motor)	19.38 ± 11.79	2-56
LEDD	452.07 (292.45)	0-1262
MoCA	25.09 (2.95)	12-30
HAM-A	13.84 (10.14)	0-35
HAM-D	12.97 (8.97)	0-35
PAS total	11.57 (8.27)	0-42
PAS-A	6.69 ± 4.59	0-20
PAS-B	2.41 ± 2.68	0-11
PAS-C	2.43 ± 2.71	0-12
PDSAI	15.43 ± 9.68	0-37

	N%
Education
Primary school	14 (9%)	
Grade 10	25 (15%)
High school	43 (25%)
Undergraduate degree	65 (38%)
Postgraduate degree	19 (12%)
Gender (N%)
Male	126 (57%)
Female	46 (43%)
HY
1	47.7%
2	37.4%
3	7.5%
Anxiety diagnosis	33 (19%)
Depression diagnosis	78 (45%)

*Note.* UPDRS = Unified Parkinson’s Disease Rating Scale; HY = Hoehn & Yahr; LEDD = Levodopa Equivalent Daily Dose; MoCA = Montreal Cognitive Assessment; HAM-A = Hamilton Anxiety Rating Scale; HAM-D = Hamilton Depression Rating Scale; PAS: Parkinson’s Anxiety Scale - PDSAI = Parkinson’s Disease Specific Anxiety Inventory.

### Item Frequency

PDSAI item and domain descriptions, as well as frequencies for each item, are provided in Table 3. Based on item response frequency, there were no ceiling or floor effects detected. A visual depiction of item frequencies is provided in Figure 2.

**Table 3.** Item Frequency (%, N) for Total Sample (N = 172).

#	Item	Domain	% Yes	N Yes	% Missing	N Missing
1	Acceptance	Disease-related coping	36	62	0	0
2	Adjustment	Disease-related coping	36	62	0	0
3	Worry about diagnosis	Disease-related coping	45	77	1	1
4	Future uncertainty	Disease-related coping	53	91	0	0
5	Motor – tremor, walking, balance	Motor	48	83	0	0
6	Current “off” times	Treatment	38	65	3	5
7	Future “off” times	Treatment	40	68	2	4
8	Current dyskinesia	Treatment	43	74	0	0
9	Future dyskinesia	Treatment	48	88	1	1
10	Future social events	Disease-related coping	38	66	0	0
11	Extra medication in public	Disease-related coping	34	58	1	1
12	Social isolation	Disease-related coping	31	53	0	0
13	Fear of falling	Motor	40	68	0	0
14	Fear of losing control	Non-motor	37	63	0	0
15	Current family burden	Disease-related coping	43	74	0	0
16	Future family burden	Disease-related coping	52	90	0	0
17	Cognitive decline	Cognitive	51	87	0	0
18	Freezing	Motor	31	53	1	1
19	Drooling, dizziness, speech		44	75	1	1
20	Driving and travelling	Disease-related coping	38	65	1	1
21	Embarrassment from tremor, walking, balance problems	Motor	42	73	0	0
22	Embarrassment about drooling, dizziness and iADLs	Non-motor	40	69	0	0
23	Embarrassment about “off” times	Treatment	34	59	2	4
24	Embarrassment dyskinesias	Treatment	36	62	1	1
25	Embarrassment about cognitive decline	Cognitive	42	72	0	0
26	Avoidance due to symptoms	Motor	42	72	0	0
27	Avoidance due to symptoms	Non-motor	36	62	1	2
28	Avoidance due to off time	Treatment	34	58	3	5
29	Avoidance due to dyskinesia	Treatment	36	62	1	2
30	Avoidance due to cognitive impairment	Cognitive	40	68	0	0
31	Frustration about symptoms	Motor	47	81	0	0
32	Frustration about symptoms	Cognitive	47	80	1	1
33	Agitation, restlessness	Non-motor	40	68	1	1
34	Internal tremor	Non-motor	43	74	2	3
35	Sleep	Non-motor	47	80	0	0
36	Anxiety increases tremors	Non-motor	56	97	0	0
37	ICDs	Treatment	33	57	1	1
38	ICD management	Treatment	29	49	2	4
39	Pre DBS	Treatment	30	52	1	1
40	Post DBS	Treatment	15	25	1	1

**Figure 2.**
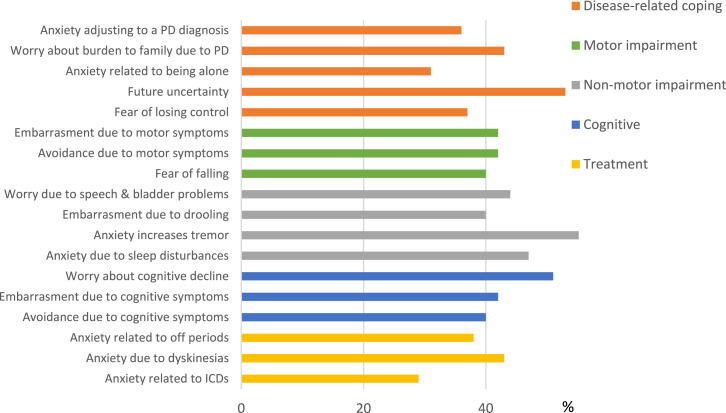
Item frequencies (%Yes) for a selection of PD-specific symptoms from the PDSAI.

### Reliability and Validity

Results of the reliability and validity analyses are conveyed in Tables 4 and 5. The PDSAI showed strong internal consistency and convergent validity with parallel anxiety measures.

**Table 4.** Correlation Results of the PDSAI Reliability Analysis.

Reliability	*r*	N
Cronbach’s alpha	0.93	153
Split-half	0.68	153

**Table 5.** Correlation Results of the PDSAI Validity Analysis.

Validity	Measure	*r*	N
Convergent	HAM-A	.508*	148
	PAS total	.747*	110
	PAS-A	.597*	111
	PAS-B	.618*	111
	PAS-C	.702*	111
	LSAS	.697*	80, AUS sample only
Divergent	MoCA	.107 (*P* = .195)	148
	HAM-D	.553*	148
	SAS	.453*	83, AUS sample only

*P < .001. *Note*. HAM-A = Hamilton Anxiety Scale; PAS = Parkinson’s Anxiety Scale; LSAS = Liebowitz Social Anxiety Scale; MoCA = Montreal Cognitive Assessment; HAM-D = Hamilton Depression Rating Scale; SAS = Starkstein Apathy Scale.

## Discussion

There are currently no measures which gauge PD-specific triggers and manifestations of anxiety. The PDSAI is the first inventory of its kind, capturing the unique sources of anxiety for people with PD. More so than other anxiety scales, the PDSAI directly informs behavioural interventions designed to lower anxiety via the precise identification of relevant treatment targets. It may also serve as a quick screening tool to be used during routine neurology visits and may help to standardize communication about anxiety presentation in PD, to facilitate coordination of care across various medical specialties.

The PDSAI may also allow for additional innovations in PD anxiety research. For example, it may provide for an improved clinical trial outcome measure for primary anxiety treatment trials in PD and an improved secondary outcome measure for studies of other aspects of PD care. It may also be specific enough for use in conjunction with neuroimaging studies to identify unique biological markers of PD anxiety.^12,26^

Frequency analyses revealed that the greatest sources of concern for people with PD were related to future insecurity, cognitive decline, future family burden, and tremor provoked by anxiety. Psychometric properties revealed high internal consistency and strong construct validity of the inventory, particularly in terms of convergent validity with the PAS.^17^ High concordance between the PDSAI and PAS indicates that both measures tap into the same underlying construct of PD anxiety. However, the PDSAI provides additional contextual information not captured by the PAS.

Of note, the PDSAI is not designed to replace the existing PAS, but rather to complement it, by providing additional information about the unique triggers and manifestations of anxiety in PD. While the PAS is an effective rating scale for measuring anxiety severity and type (persistent, episodic, avoidance), it provides little information on the content and context of the PD specific anxiety triggers. The PDSAI is designed to fill this gap, in order to further inform personalized treatment plans and interventions. For example, the individualized assessment of anxiety triggers specific to each PwP yields data that can be used to guide psychoeducation, cognitive restructuring, and exposure based interventions in the context of cognitive behavioral therapy.

Moreover, a recent systematic review highlighted the importance of identifying triggers of anxiety for people with PD to maximise the efficacy of psychotherapy interventions.^10^ Given the complexity of anxiety in PD and its many manifestations,^4^ interventions tailored to PD-specific triggers target the appropriate source of the anxiety and allow for person-centred care. We previously used this approach, finding that personalised Cognitive Behavioural Therapy reduced anxiety for people with PD post-therapy and a three and six month follow ups, with additional benefits of reduced carer burden.^9^ The tailored therapeutic approach may even benefit proposed virtual reality assisted psychotherapy for PD, where treatment involves specific scenarios relevant to the triggers of people with PD.^27^

A strong correlation between the PDSAI and LSAS was also observed as expected, given its targeted assessment of embarrassment related to PD symptoms and avoidance behaviours. While the PDSAI does not measure somatic symptoms of anxiety, a reduced, yet still significant association between the PDSAI and the HAMA was observed. The moderate correlation between the PDSAI and the HAMD is not surprising, given the high rate of comorbidities between depressive and anxiety disorders in PD. Of note, measures such as the HAM-A and HAM-D which heavily target the somatic symptoms may complicate assessment given the overlap of the somatic symptoms of anxiety and depression and the physical symptoms of PD.^4^ However, these two scales were used as they are gold-standard “clinician rated” measures of anxiety and depression in PD, recommended for use by NIH Common Data Elements Project.

Of note, higher scores on the PDSAI were correlated with higher severity of anxiety across several measures. Therefore, it could be inferred that the severity of anxiety would increase with higher number of triggers reported. Alternatively, it is possible that PWP with more severe anxiety are more impacted by the effects of daily life stressors and PD symptom fluctuations. It should also be noted that the inventory is not intended for use as a rating scale, and therefore does not have a cut-off or threshold for identifying severity levels of anxiety in PD. However, exploratory analyses did reveal that the mean PDSAI score was significantly higher in people with PD with any DSM-V diagnosis of an anxiety and/or depressive disorder compared to those without a DSM-V diagnosis of anxiety or depression.

In summary, the PDSAI was designed to be used in conjunction with other established measures of anxiety in PD to better characterize the clinical picture. Despite its pernicious effects, anxiety is under-diagnosed and sub-optimally treated in clinical practice. Screening tools designed to assess unique manifestations of anxiety in PD are required to improve anxiety detection rates and increase rates of mental health referral and treatment utilization. Routine anxiety screening as part of standard PD care also affords providers the opportunity to frame anxiety as common and treatable symptom of PD, in order to reduce stigma and improve awareness of the neuropsychiatric challenges inherent in PD.

### Limitations and Future Directions

Although combining data from two study centres (Australia and USA) boosted our sample size, we did not reach our target 400 participants (10 participants per item). Only 172 participants were enrolled. We therefore were unable to conduct itemized reductions using factor analysis statistical methodology. In addition, our overall sample was predominantly White and our US sample comprised only males due to the study being conducted in the VA health care system, which tends to be a heavily male cohort. As such, the possibility of a short form PDSAI will be explored in a larger, more diverse, international multicentre study.

We did not formally track PDSAI completion time. The PDSAI was administered as a self-report instrument alongside the self-report PAS. This did not impact item completion of the PDSAI, as evidenced by the low percentage of missing data. It is also an indicator of the ease of use of the instrument. We estimate that the PDSAI will take approximately 10-15 minutes to complete.as a stand-alone screening tool. Moreover, to reduce participant burden, we limited the number of questionnaires administered in this study. Future research will explore the relationship between the PDSAI and other self-report measures of anxiety, like the Geriatric Anxiety Inventory (GAI).

It is also important to note that PDSAI was not designed for use as a diagnostic tool, but rather as an inventory to capture self-reported PD-specific triggers of anxiety. For this reason, we did not test discriminant validity between the PDSAI and DSM-V diagnostic criteria. Nevertheless, given the robust completion rates and low number of itemized missing data, the analysis provided indicates satisfactory reliability and validity of the new PDSAI tool. We did not assess all clinometric properties of the PDSAI, such as its sensitivity to change, which will require longitudinal assessments. However, our recent pilot study examining telehealth mindfulness based cognitive behavioural therapy used the PDSAI as an outcome measure and observed significant changes in the PDSAI (with large effect sizes) over time.^28^ Sensitivity to change will continue to be explored in future studies.

## Conclusion

The PDSAI is the first inventory to identify the specific and heterogeneous triggers of anxiety unique to PD.^2^ A patient-reported outcome such as this may facilitate the longitudinal assessment of anxiety in clinical research and assist with detection and of management in practice settings. This will not only positively impact quality of life for patients and families through improved understanding and diagnosis of anxiety, but it will also have a significant impact on all aspects of PD care.
